# Postnatal relative adrenal insufficiency results in methylation of the glucocorticoid receptor gene in preterm infants: a retrospective cohort study

**DOI:** 10.1186/s13148-018-0497-9

**Published:** 2018-05-18

**Authors:** Masato Kantake, Natsuki Ohkawa, Tomohiro Iwasaki, Naho Ikeda, Atsuko Awaji, Nobutomo Saito, Hiromichi Shoji, Toshiaki Shimizu

**Affiliations:** 1 Neonatal Medical Center, Juntendo University Shizuoka Hospital, 1192 Nagaoka, Izunokuni, Shizuoka, 410-2295 Japan; 2 Division of Pediatrics, Juntendo University School of Medicine, 3-1-3, Hongo, Bunkyo, 113-8421 Japan

**Keywords:** Adrenal insufficiency, Chronic lung disease, Circulatory collapse, Epigenetics, Glucocorticoid administration, Glucocorticoid receptor gene, Intrauterine growth restriction, HPA axis, Methylation, NR3C1

## Abstract

**Background:**

To investigate the relationship between early-life stress and glucocorticoid receptor (GR) gene methylation, which may result in long-lasting neurodevelopmental impairment, we performed a longitudinal analysis of the methylation ratio within the GR gene promoter 1F region using next-generation sequencing in preterm infants.

Cell-free DNA was extracted from the frozen serum of 19 preterm birth infants at birth and at 1 and 2 months after birth. All were admitted to the neonatal intensive care unit of Juntendo University Shizuoka Hospital between August 2014 and May 2016 and suffered from chronic lung disease (CLD).

Through bisulfite amplicon sequencing using an Illumina Miseq system and Bismark-0.15.0 software, we identified the rate of cytosine methylation.

**Results:**

Patients’ sex and body weight standard deviation were extracted as the associated independent variables at birth. Sex, glucocorticoid administration for treating CLD, and postnatal invasive procedures (surgical operation and blood sampling) were extracted as the associated independent variables at 1 month. Methylation rates increased significantly between postnatal 1 and 2 months at 9 of the 39 CpG sites. Postnatal glucocorticoid administration to treat circulatory collapse was the most-associated independent variable with a positive regression coefficient for a change in methylation rate at these nine CpG sites. It also influenced the methylation ratio at 22 of the 39 CpG sites at 2 months of age. The standard deviation (SD) score at birth was extracted as an independent variable, with a negative regression coefficient at 9 of the 22 CpG sites together with glucocorticoid administration.

**Conclusions:**

The results of this study indicate that a prenatal environment that results in intrauterine growth restriction and postnatal relative adrenal insufficiency requiring glucocorticoid administration leads to GR gene methylation. That, in turn, may result in neurodevelopmental disabilities.

## Background

The improved survival of babies at early gestational ages (GAs) is considered a success of modern neonatology. In the current post-steroid and post-surfactant era of neonatology, extremely preterm babies who survive have non-negligible rates of neurological disabilities such as cerebral palsy, mental deficits, sensorineural impairment, and cognitive dysfunction ranging from mild to severe [1, 2]. Infants born with a birth weight < 10th percentile or small for gestational age (SGA) [3, 4] and infants with chronic lung disease (CLD) characterized by prolonged inflammation of lung tissue are at increased risk for neonatal mortality, and preterm infants suffer from both short- and long-term morbidities [5, 6].

The most commonly implicated mechanism of these long-term effects is the dysregulation of the hypothalamus–pituitary–adrenal (HPA) axis. Indeed, dysregulation of this axis has been noted in extremely low birth weight and very low birth weight (VLBW) survivors across their lifespan [7, 8]. An impaired HPA axis is an important risk factor for inflammatory disease, somatic fatigue, pain disorders, and psychiatric conditions such as depression and post-traumatic stress disorder [9, 10]. Activity of the HPA axis is regulated by the hypothalamic glucocorticoid receptor (GR) encoded by the nuclear receptor subfamily 3 group C member 1 (*NR3C1*) gene, which mediates a negative feedback loop [11].

Promoter DNA methylation is a well-established epigenetic regulator of gene expression. Prenatal stressors, such as maternal depression and anxiety and maternal exposure to stressors, are associated with GR gene methylation [12, 13]. Similarly, adults who retrospectively report a history of childhood maltreatment, early parental death, and childhood trauma show associations with GR gene methylation [14–16]. These links have also been demonstrated in postmortem brains from adult suicides [17] as well as from patients who suffer from depression or bipolar disorder [18]. In our previous study, we showed that a postnatal environment that includes the need for acute care and prolonged physical separation under neonatal intensive care affects epigenetic programming of GR expression through methylation of the *NR3C1* promoter in premature infants, which might result in glucocorticoid resistance later in life [19]. These studies were performed by using several tissues, such as placenta [12], cord blood [13], peripheral blood [14, 19], saliva [15, 16, 18], and brain [17].

Several important regions are known to exist in the 39 CpG sites analyzed in the present study [20]. Among them, CpG 30–32 are the most important regions known to be binding sites for nerve growth factor inducible protein A (NGFI-A), which has emerged as a central regulator of early inflammatory and immune processes and potentiates GR 1-F promoter activity. The methylation rate in this region is influenced by an adverse environment during early life [17]. The high methylation status in this region is also known to result in low GR mRNA expression in an animal model in vitro [21]. CpG 35 has been reported to be associated with maternal stress, a low level of prenatal care, and childhood maltreatment. CpG 36 has been associated with maternal psychopathology during pregnancy. CpG 37 methylation has been associated with both early-life stress and maternal psychopathology. CpG 39 methylation has been associated with childhood maltreatment and prenatal stress. However, CpG site-specific methylation findings for CpG 1–29 are scarce.

Recently, Giarraputo et al. showed that the GR gene methylation in infants in neonatal intensive care unit (NICU) is correlated with high medical risk which is defined by many medical variables (Neonatal Therapeutic Intervention Scoring System (NTISS)) [22].

To investigate the relationship between the early-life environment and GR gene (*NR3C1*) methylation status, which may take part in long-lasting neurodevelopmental impairment, we performed a longitudinal analysis of methylation ratios within the GR gene promoter 1F region using next-generation sequencing in preterm infants with CLD. In this study, we used cell-free DNA which is thought to be derived from proliferating/apoptotic lymphocytes from the participant’s peripheral blood [23].

## Methods

This study was approved by the Juntendo University Ethics Committee and conducted according to the principles of the Declaration of Helsinki. Nineteen infants admitted to the NICU of Juntendo University Shizuoka Hospital between August 2014 and May 2016 were enrolled in this study after written informed consent had been obtained. All serum samples were routinely collected at birth and at 1 and 2 months after birth and stored at − 80 °C until analysis. The criterion for CLD was the need for additional oxygen after the age of 28 days. Cell-free DNA was extracted from 100 μL frozen serum using a DNA Extractor SP Kit according to the manufacturer’s instructions (Wako Pure Chemical Industries, Ltd. Osaka, Japan), and bisulfite-treated DNA was obtained using an EZ DNA Methylation Direct Kit (Zymo Research Corp., Irvine, CA, USA). A 5-μL aliquot of the resulting 10-μL bisulfite-treated genomic DNA solution was subjected to polymerase chain reaction analysis to amplify the GR promoter 1F region, as previously described [19]. The amplicons were purified using a gel-based clean up, and ViewaBlue Stain KANTO (Kanto Chemical Co., Inc. Tokyo, Japan) was used for DNA staining. The purified amplicons were subjected to a NEBNext Ultra II Library Preparation Kit for Illumina (New England Biolabs Japan, Inc., Tokyo, Japan) including dual indexing. These libraries were multiplexed and sequenced on an Illumina MiSeq system (Illumina Inc., San Diego, CA, USA). The reads were aligned to an in silico converted reference using Bowtie2-2.2.1, and variant calling was used to identify the percentage of methylated cytosines using Bismark-0.15.0. [24]. We analyzed the epigenetic changes in the GR promoter 1F region (containing 39 CpG sites ranging from − 3466 to − 3189 bp upstream of the ATG start site).

### Statistical analysis

We performed Wilcoxon’s signed-rank test to evaluate longitudinal differences between birth and at 1 and 2 months after birth. A stepwise multiple regression analysis was performed to investigate the relationships of methylation ratio at birth, 1 month, and 2 months with the amount of increase during months 1–2 at each CpG site as dependent variables and pre- and postnatal parameters in preterm infants as independent variables. To perform this analysis, several nominal variables were converted into categorical variables by grouping the values into two categories. The adjusted coefficient of determination (*R*)^2^ is the fraction of information of the dependent variable that is explained by the independent variables. A two-tailed *p* value < 0.05 was considered statistically significant. All of the statistical analyses were performed using SPSS v24.0 (IBM Corp., Armonk, NY, USA) software.

## Results

The abbreviations in the present study are listed and described at the end of the main text and in Table 1.

**Table 1:** Patient characteristics

	Range	Number	Mean, SE		Range	Number	Mean, SE
Sex		*M* = 12		AS	0–24	13	12, 2.43
		*F* = 7					
GA	24w3d–28w2d		27w2d, 2d	Gc circ	0–17.4	8	1.44, 0.91
BW	402–1226		825, 54.7	Gc CLD	0–38	3	2.32, 2.0
SD	− 3.95–0.17		− 1.36, 0.31	Opi	0–7	13	3.37, 0.58
dSD	− 2.3–0.97		− 0.61, 0.15	Bf	0.1–7.6	19	4.39, 0.48
Mv	1–60	19	32.4, 5.15	In	0–1.6	15	0.48, 0.10
Hc	30–84	19	57.4, 4.45	PDA		7	

*M* male, *F* female, *GA* gestational age, *BW* birth weight (g), *SD* standard deviation of body weight, *dSD* change in SD scores between 0 and 2 months, *Mv* duration of mechanical ventilation with intra-tracheal intubation between 0 and 2 months after birth (days), *Hc* heel cut procedure for blood examination between 0 and 2 months after birth (times), *AS* antenatal steroid administration (mg), *Gc circ* glucocorticoid administration between 0 and 2 months after birth as a treatment for circulatory collapse (mg/kg prednisolone), *Gc CLD* glucocorticoid administration between 0 and 2 months after birth as a treatment for or prevention of CLD (mg/kg prednisolone), *Opi* opioid administration between 0 and 2 months after birth (mg fentanyl), *Bf* breast fed volume between 0 and 1 month after birth (mL/kg) (kg was calculated by birthweight + bodyweight at 2 months/2), *In* indomethacin administration for PDA closure (mg/kg), *PDA* underwent surgery to close a patent ductus arteriosus between 0 and 2 months after birth

### Participant characteristics

Nineteen participants completed the analysis, and their characteristics are shown in Table 1. The GAs and birth weights of the infants ranged from 24 weeks + 3 days to 28 weeks + 2 days and from 316 to 1226 g, respectively. All infants received mechanical ventilation. Four were below − 2 SD of birth weight. Seven infants underwent an operation to close the patent ductus arteriosus (PDA). Twelve infants received antenatal steroid (AS) administration and 11 received postnatal steroid administration. Of these 11 infants, 9 were treated for circulatory collapse and 2 were treated for CLD. Fourteen infants received an opioid for sedation.

### Relationship between prenatal parameters and GR gene methylation at birth

We examined the associations between the methylation ratios at the 39 CpG sites in the GR 1F promoter and GA at birth (weeks), body weight SD scores at birth calculated by the Japanese standard, AS administration (mg), and sex. Methylation rates in the GR 1F region were generally low at birth. At 3 of the 39 CpG sites analyzed, only sex (male = 1, female = 0) was correlated with the methylation ratio (CpG 12: *p* = 0.046, regression coefficient, − 0.463; CpG 19: *p* = 0.027, regression coefficient, 0.505; CpG 27: *p* = 0.030, regression coefficient, − 0.497). At 4 of the 39 CpG sites analyzed, only SD scores were correlated with the methylation ratios (CpG 7: *p* = 0.048, regression coefficient, − 0.460; CpG 25: *p* = 0.040, regression coefficient, − 0.475; CpG 36: *p* = 0.047, regression coefficient, − 0.461; CpG 38: *p* = 0.002, regression coefficient, − 0.675). The methylation ratio at CpG 24 was associated only with AS administration (*p* = 0.025, regression coefficient, − 0.512) (Fig. 3).

### Relationship between prenatal and postnatal (0–1 month) parameters and GR gene methylation at 1 month

We analyzed the relationships of methylation rates at 1 month with prenatal and postnatal (0–1 month) parameters (sex, GA, SD, AS, Hc, Bf, Gc circ, Gc CLD, Opi, dSD, In, and PDA) at the 39 CpG sites. The results are shown in Fig. 3. Briefly, postnatal parameters such as glucocorticoid administration for CLD, surgical operation, or heel-cut blood sampling were extracted as independent variables with positive regression coefficients in CpG 5, 8, 14, 25, 32, 33, 34, 35, and 38, except that sex in CpG 19 had a negative regression coefficient.

### Longitudinal changes in methylation status

**Fig. 1:**
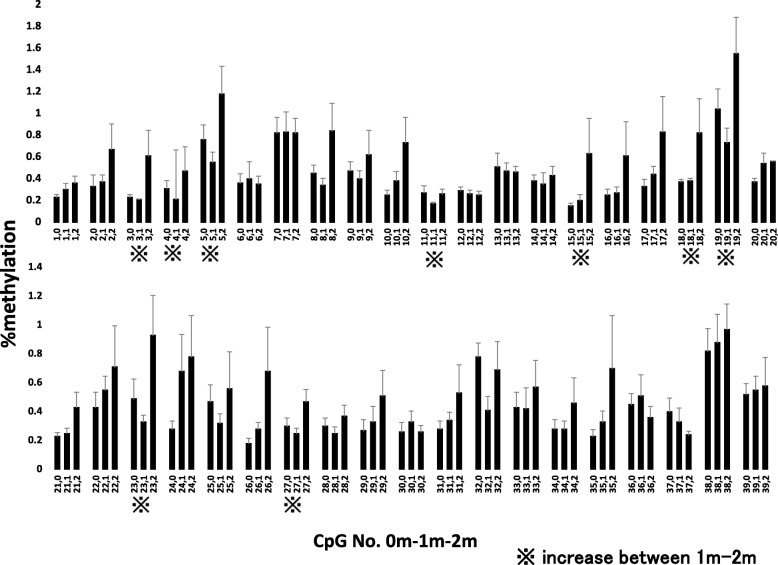
Longitudinal analysis of glucocorticoid receptor (GR) 1F promoter methylation. Methylation ratios at birth and at 1 and 2 months after birth in the 39 CpG sites analyzed are shown. The left, center, and right triplet bars indicate the methylation rates at birth, 1 month, and 2 months, respectively, with standard error bars. The asterisks under the CpG numbers indicate the methylation rates at CpG sites that significantly increased at between 1 and 2 months after birth

Methylation rates between birth and postnatal 1 month were stable at all of the 39 CpG sites examined. By contrast, methylation rates significantly increased from postnatal 1 to 2 months at 9 of the 39 CpG sites (CpG 3: *p* = 0.043; CpG 4: *p* = 0.047; CpG 5: *p* = 0.031; CpG 11: *p* = 0.014; CpG 15: *p* = 0.038; CpG 18: *p* = 0.047; CpG 19: *p* = 0.005; CpG 23: *p* = 0.0025; CpG 27: *p* = 0.03) (Fig. 1).

### Relationships of the change in methylation ratio during months 1 and 2 with prenatal and postnatal parameters between birth and 2 months of age

We analyzed the relationship between changes in the methylation rates at CpG 3, 4, 5, 11, 15, 18, 19, 23, and 27, when significant increases in methylation rates were observed during months 1–2 after birth, and the prenatal and postnatal environments at 0–2 months of age. The independent variables analyzed at 0–2 months were sex, GA, SD, AS, days of mechanical ventilation (Mv), number of heel-cut (Hc) procedures, breast milk intake (mL/kg) (breast-fed [Bf] baby), amount of glucocorticoid administered (mg/kg prednisolone) for treatment of circulatory collapse (Gc circ), and CLD (Gc CLD), amount of opioid (Opi) administered (times 0.1 mL fentanyl), change in body weight SD scores (dSD), amount of indomethacin (In) administered (mg/kg), and experience of receiving surgery to close a patent ductus arteriosus (PDA) (yes = 1, no = 0).

At seven of the nine CpG sites, Gc circ was solely extracted as an efficient independent variable with a positive regression coefficient. CpG 3 (Gc circ [regression coefficient, 0.885, overall *p* < 0.001, adjusted *R*^2^ = 0.771]); CpG 4 (Gc circ [regression coefficient, 0.871, overall *p* < 0.001, adjusted *R*^2^ = 0.744]); CpG 5 (Gc circ [regression coefficient, 0.687, overall *p* = 0.001, adjusted *R*^2^ = 0.440]); CpG 15 (Gc circ [regression coefficient, 0.895, overall *p* < 0.001, adjusted *R*^2^ = 0.790]); CpG 18 (Gc circ [regression coefficient, 0.926, overall *p* < 0.001, adjusted *R*^2^ = 0.848]); CpG 19 (Gc circ [regression coefficient, 0.689, overall *p* = 0.001, adjusted *R*^2^ = 0.444]); and CpG 23 (Gc circ [regression coefficient, 0.800, overall *p* < 0.001, adjusted *R*^2^ = 0.619]). None of the eight parameters examined was extracted as an efficient independent variable at the remaining two CpG sites (CpG 11 and CpG 27).

### Relationship between methylation rate at postnatal 2 months and prenatal and postnatal parameters

**Fig. 2:**
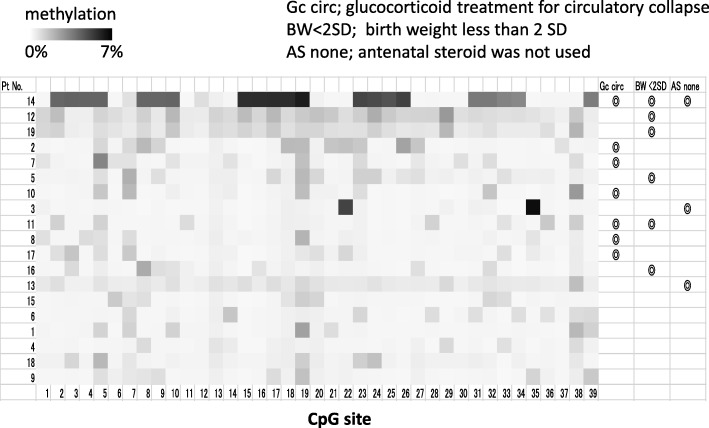
Heat map of GR 1F promoter methylation at 2 months old. Patients who received a glucocorticoid for circulatory collapse (Gc circ), whose bodyweights were less than − 2 SD (BW < − 2 SD), and who were not administered an antenatal steroid (AS) are marked by the double circle in the right area of the heat map

Next, we analyzed the relationship between methylation rates at 2 months after birth and prenatal and postnatal parameters, e.g., sex, GA, SD, AS, Hc, Bf, Gc circ, Gc CLD, Opi dSD, In, and PDA at all of the 39 CpG sites. The results are shown in Table 2. Briefly, some independent variables were extracted from 24 of the 39 CpG sites examined. Gc circ was extracted as an independent variable at 22 CpG sites with a positive regression coefficient. The volume of breast milk consumed was extracted as the sole independent variable at the remaining two CpG sites, with a negative regression coefficient (CpG 11 and CpG 36). SD scores at birth were simultaneously extracted as independent variables at 7 of 22 sites at which Gc circ was extracted, with a negative regression coefficient. AS administration was extracted as an independent variable at 3 of the 22 sites, with a negative regression coefficient. Glucocorticoid administration to treat CLD was extracted as an independent variable at CpG 2 with a negative regression coefficient and at CpG 8 with a positive regression coefficient. Figure 2 is a heat map of the methylation status focused on the effect of three major independent variables (Gc circ, AS, and SD).

**Table 2:** The relationships between methylation status at 2 months and pre- and postnatal environments

CpG no.	2	3	4	5	8	9	10	11
Gc circ	0.568	0.877	0.968	0.754	0.715	0.855	0.66	
SD	− 0.547						− 0.352	
Gc CLD	− 0.365				0.327			
AS	− 0.193				− 0.298	− 0.254		
Bf								− 0.492
Adjusted *R*^2^	0.922	0.774	0.933	0.544	0.752	0.858	0.724	0.198
Overall *p*	^※^	^※^	^※^	^※^	^※^	^※^	^※^	0.032
CpG no.	12	15	16	17	18	19	23	24
Gc circ	0.802	0.789	0.837	0.755	0.887	0.842	0.894	0.639
SD		− 0.255	− 0.234	− 0.285				− 0.364
Gc CLD								
Bf								
Adjusted *R*^2^	0.623	0.838	0.909	0.811	0.89	0.691	0.788	0.702
Overall *p*	^※^	^※^	^※^	^※^	_※_	^※^	^※^	^※^
CpG no.	25	26	31	32	33	34	36	39
Gc circ	0.81	0.865	0.908	0.877	0.892	0.902		0.827
SD	− 0.246							
Gc CLD								
AS								
Bf							− 0.506	
Adjusted *R*^2^	0.869	0.734	0.814	0.756	0.784	0.803	0.213	0.665
Overall *p*	^※^	^※^	^※^	^※^	^※^	^※^	0.027	^※^

Regression coefficients of independent variables are shown. A blank means not extracted as independent variables

*Gc circ* glucocorticoid administration for circulatory collapse, *SD* SD score of birth weight, *Gc CLD* glucocorticoid administration for CLD, *AS* antenatal glucocorticoid administration, *Bf* amount of human milk

^※^*p* < 0.001

## Discussion

This is the first longitudinal report to demonstrate that prenatal and postnatal environments induce gene methylation. The results of this study show that postnatal Gc circ had positive and strong effects on GR gene methylation at 1–2 months of age, which may result in neurodevelopmental disability in later life due to dysfunction in the HPA axis. Simultaneously, prenatal factors such as SD scores at birth and antenatal glucocorticoid administration had moderate negative effects on GR gene methylation.

**Fig. 3:**
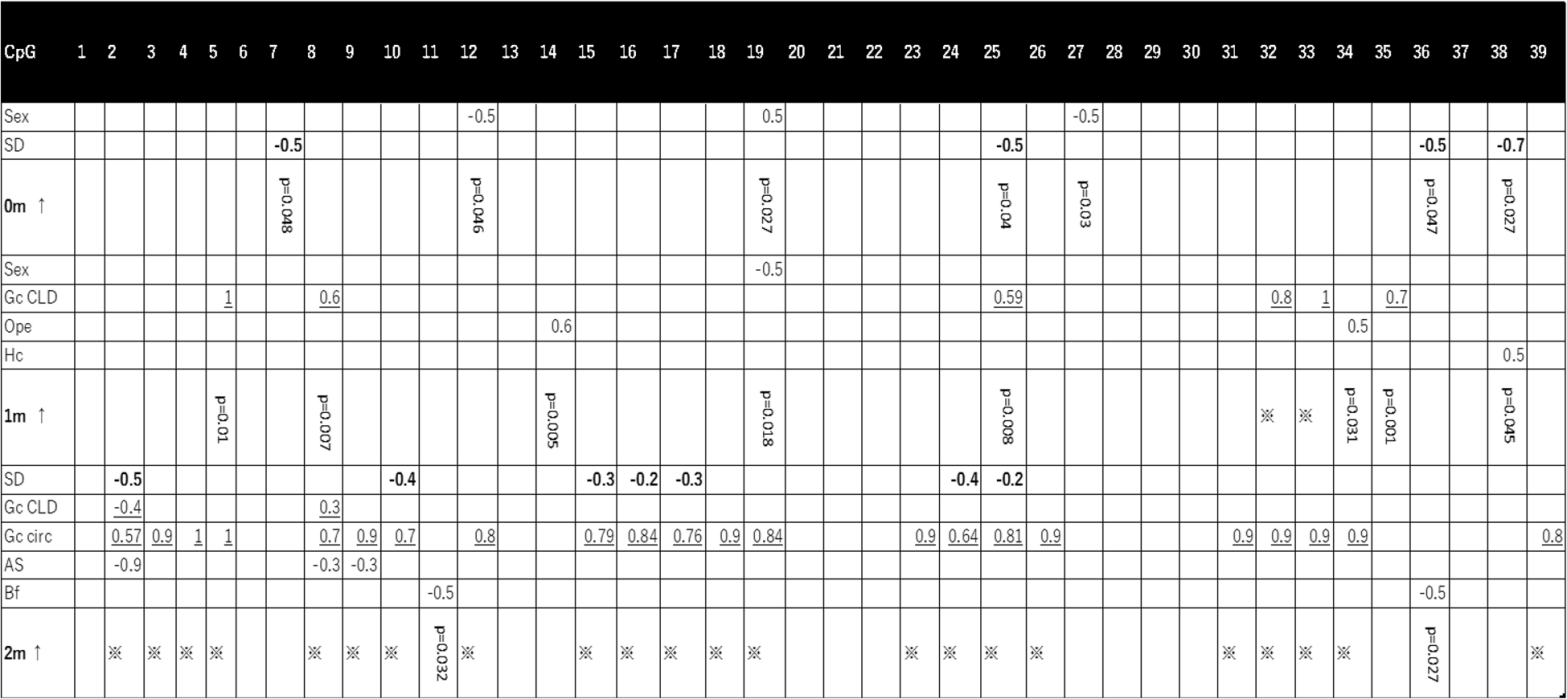
Extracted independent variables in the 39 CpG sites at birth (0 m), 1 month (1 m), and 2 months (2 m). Regression coefficients of the SD scores are bolded and those of glucocorticoid administration are underlined

In the present study, glucocorticoid administration at 0–1 month influenced GR gene methylation at 1 month (Fig. 3). Our criteria for using glucocorticoid for CLD are as follows: mean airway pressure of mechanical ventilation > 10 mmHg and FiO_2_ > 0.4, which indicate a severe lung condition due to relative adrenal insufficiency.

Neonatal hypotension is commonly seen in premature neonates, and its incidence is inversely related to GA at delivery. Hypotension in preterm infants is predominantly due to either abnormal peripheral vasoregulation or myocardial dysfunction. Another etiology of hypotension in this population is physiological factors, including the presence of relative adrenal insufficiency secondary to an immature HPA axis [25]. Ng et al. showed an increase in plasma adrenocorticotrophic hormone with the use of inotropes and volume expanders in VLBW infants with hypotension [26]. In addition, low serum cortisol levels were also found in these patients [27]. These findings suggest that the adrenal cortex may be the primary site of dysfunction in this patient population, and not the hypothalamus or pituitary gland. Overall, these findings imply a normal response of the pituitary gland to hypotension, but the adrenal glands are transiently unable to maintain cortisol secretion during the immediate postnatal period. Hydrocortisone is the recommended treatment for hypotensive neonates if volume resuscitation with isotonic saline and dopamine treatment (10 μg/kg/min) are unsuccessful [28]. Our strategy to treat neonatal hypotension, which is defined as a mean blood pressure lower than the neonate’s GA, is described above. We also confirmed the effects of glucocorticoids by improvements in blood pressure and urine output soon after administration. We conclude that the hypotension observed in our study population resulted from relative adrenal insufficiency.

Postnatal steroids are widely used to manage preterm infants with CLD. Several reports have indicated that infants with CLD may develop adrenal insufficiency during the first 1–2 weeks of life [26, 29]. All of the infants in this study developed CLD, implying that they may have had some degree of adrenal insufficiency.

Taken together, these findings led us to hypothesize that severe adrenal insufficiency followed by both CLD and circulatory collapse results in GR gene methylation. Adrenal insufficiency during the early stage of life results in upregulation of the HPA axis later in life to respond adequately to stress.

Antenatal glucocorticoid administration had a moderate negative effect on GR gene methylation. In contrast, postnatal glucocorticoid administration for CLD and circulatory collapse had a positive effect on GR gene methylation. Thus, glucocorticoid demand due to relative adrenal insufficiency, not glucocorticoid administration itself, may result in GR gene methylation.

SD scores at birth were related to GR gene methylation at birth and at 2 months of age. Notably, the CpG sites affected at 2 months by SD (CpG 10, 15, 16, 17, 24, and 25) were different from those affected at birth by SD (CpG 7, 25, 36, and 38), except CpG 25. All of the SD effects at birth had disappeared by 1 month, even those in CpG 25. In addition, SD was always affected together with postnatal glucocorticoid administration only at 2 months. Thus, it is possible that the long-lasting influence of low SD (e.g., intrauterine growth restriction) on GR gene methylation is indirect.

Permanent changes in gene expression have been observed in multiple genes as a consequence of SGA. Dysregulation of the epigenome may explain changes that are propagated from parent to daughter cells in SGA offspring throughout life [30]. The results of this study suggest that dysregulation of several genes induced by intrauterine growth restriction may increase susceptibility to circumstances after birth, particularly relative adrenal insufficiency, leading to GR gene methylation, which in turn results in neurodevelopmental disabilities. Stressful postnatal procedures, such as the Hc procedure or Ope, were correlated with significant positive regression coefficients at three CpG sites at 1 month. Taken together, these findings suggest that relative adrenal insufficiency, which leads to severe CLD and circulatory collapse and the inability to respond to a stressful postnatal environment in the NICU, may result in GR gene methylation at 2 months after birth.

In this study, well-known CpG sites, which are methylated by early-life stress directly, such as CpG 30–32, CpG 35–37, and CpG 39, were affected at 2 months almost only by Gc circ. In contrast, CpG 1–29, which are not well-known as sites methylated by early-life stress, were affected by Gc circ and low SD. CpG 1–29 may be methylated by both the intrauterine and the postnatal environment. Figure 2 shows that CpG 30–39 and CpG 1–29 are widely methylated, which may result in a change in three-dimensional DNA structure.

It should be noted that the volume of mothers’ milk fed to infants during the first 2 months after birth had a weak but significant negative effect on GR methylation (CpG 11 and 36). Maternal breastfeeding has been emphasized as an influential factor in early childhood development [31]. However, empirical evidence for the effects of breastfeeding on children’s cognitive development has been conflicting. A comprehensive review by the Agency for Healthcare Research and Quality summarized 400 articles and found that breastfeeding had few or small effects on children’s cognitive ability [32]. This study may provide new insights into the effects of breastfeeding.

Because epigenetic modifications occur in a tissue-specific manner, it remains unclear whether DNA methylation measured in blood reflects DNA methylation patterns in other tissues including the brain. We used cell-free DNA, which is thought to be derived from proliferating/apoptotic lymphocytes in the participant’s peripheral blood [23].

Although research conducted on the effects of early-life stress on methylation of the alternate GR promoter 1F in blood, brain, saliva, and placenta produced similar results [12–19, 33], it should be noted that the correlation of methylation between blood and brain is still controversial. Watson et al. showed that only 7.9% of CpG sites were statistically significant, showing a large correlation between blood and brain tissue [34]. Tylee et al. reviewed seven studies comparing patterns of DNA methylation between the blood and brain and suggested that CpG island methylation levels are generally highly correlated (*r* = 0.90) between them [35].

This study has some limitations. First, cell type and tissue-specific diversity of methylation of the GR gene are noted problems. This study did not include a control for variations in cell type. Second, the participants were homogeneous in race, limiting generalizability of the findings. Another limitation is the lack of information about endogenous glucocorticoid secretion. Further investigations including a longitudinal follow-up study and cell type-specific investigation will enhance our understanding of the relationships between early-life experiences and long-lasting neurodevelopmental disability.

## Conclusions

This study revealed that adverse pre- and postnatal environments, such as intrauterine growth restriction and postnatal relative adrenal insufficiency, increased glucocorticoid receptor gene methylation. This, in turn, may result in neurodevelopmental disabilities.

## Abbreviations

CLD: Chronic lung disease
GR: Glucocorticoid receptor
HPA: Hypothalamus–pituitary–adrenal gland
SGA: Small for gestational age

## Specific abbreviations in the patients’ background and perinatal factors

AS: Antenatal steroid administration (mg)
Bf: Breast fed volume between 0 and 1 month after birth (mL/kg) (kg was calculated by birthweight + bodyweight at 2 months/2)
BW: Birth weight (g)
dSD: Change of SD scores between 0 and 2 months
F: Female
GA: Gestational age
Gc circ: Glucocorticoid administration between 0 and 2 months after birth for treatment of circulatory collapse (mg/kg prednisolone)
Gc CLD: Glucocorticoid administration between 0 and 2 months after birth for treatment or prevention of CLD (mg/kg prednisolone)
Hc: Heel cut procedure for blood examination between 0 and 2 months after birth (times)
In: Indomethacin administration for PDA closure (mg/kg)
M: Male
Mv: Duration of mechanical ventilation with intra-tracheal intubation between 0 and 2 months after birth (days)
Opi: Opioid administration between 0 and 2 months after birth (mg fentanyl)
PDA: Experience of receiving surgery to close a patent ductus arteriosus between 0 and 2 month after birth
SD: Standard deviation score of body weight
